# Contributory roles of two l-lactate dehydrogenases for l-lactic acid production in thermotolerant *Bacillus coagulans*

**DOI:** 10.1038/srep37916

**Published:** 2016-11-25

**Authors:** Lifan Sun, Caili Zhang, Pengcheng Lyu, Yanping Wang, Limin Wang, Bo Yu

**Affiliations:** 1 CAS Key Laboratory of Microbial Physiological and Metabolic Engineering, Institute of Microbiology, Chinese Academy of Sciences, Beijing 100101, China; 2 School of Food Engineering and Biological Technology, Tianjin University of Science & Technology, Tianjin 300457, China; 3 College of Life Science and Bioengineering, Beijing University of Technology, Beijing 100124, China

## Abstract

Thermotolerant *Bacillus coagulans* is considered to be a more promising producer for bio-chemicals, due to its capacity to withstand harsh conditions. Two L-lactate dehydrogenase (LDH) encoding genes (*ldh*L1 and *ldh*L2) and one D-LDH encoding gene (*ldh*D) were annotated from the *B. coagulans* DSM1 genome. Transcriptional analysis revealed that the expression of *ldh*L2 was undetectable while the *ldh*L1 transcription level was much higher than that of *ldh*D at all growth phases. Deletion of the *ldh*L2 gene revealed no difference in fermentation profile compared to the wild-type strain, while *ldh*L1 single deletion or *ldh*L1*ldh*L2 double deletion completely blocked L-lactic acid production. Complementation of *ldh*L1 in the above knockout strains restored fermentation profiles to those observed in the wild-type strain. This study demonstrates *ldh*L1 is crucial for L-lactic acid production and NADH balance in *B. coagulans* DSM1 and lays the fundamental for engineering the thermotolerant *B. coagulans* strain as a platform chemicals producer.

Lactic acid has been produced by microbial fermentation for many years, and is used primarily in the food, pharmaceutical, cosmetic, and chemical industries^1^. It is also becoming a bulk building block for chemical production of the green polymer poly-lactic acid (PLA) that could be applied in bio-plastics^2^. A major advantage of microbial production of lactic acid over its chemical synthesis is that the enzyme-catalyzed microbial production results in a significantly higher optical purity^1^. Since only optically pure L- and D-lactic acid monomers could be used as PLA precursors, microbial fermentation is the preferred process for lactate production^3^,^4^.

Bacteria, fungi of the *Rhizopus* genus, yeast, cyanobacteria and various genetically modified strains have the ability to produce lactic acid^5^,^6^. Recently, several studies have shown that lactic-acid-producing thermophiles might have substantial advantages over the traditionally used mesophilic strains, such as *Lactococcus lactis* and *Lactobacillus rhamnosus*. Not only do these organisms have general advantages in terms of fermentation at high temperature (>50 °C), but can also utilize inexpensive carbon resources, require less complex nitrogen sources, and in most cases require no aeration^7^,^8^,^9^. Thermophilic *Bacillus coagulans* is one such microorganism displaying a number of these characteristics. Additionally, the ability of thermophilic *B. coagulans* to produce high levels of optically pure L-lactic acid at 50–55 °C is expected to minimize contamination in industrial-scale fermentations under non-sterile fermentation conditions, making it beneficial as an industrial strain and improving the commercial competitiveness of lactic acid production^10^,^11^.

L-Lactate dehydrogenase (L-LDH; EC 1.1.1.27) and D-lactate dehydrogenase (D-LDH; EC 1.1.1.28) are responsible for the conversion of pyruvic acid (produced by glycolysis) to L- and D-lactic acid, respectively^12^,^13^,^14^. Studies have shown that the relative catalytic efficiencies of *ldhL*- and *ldhD*-encoded products are crucial for the optical purity of lactic acid produced by *Lactobacillus* strains^3^. We previously showed that only L-LDH activity was detected in *B. coagulans* 2–6 (a L-lactic acid producer) under native conditions, and that *ldhL* transcription was much higher than that of other lactic-acid-metabolism-related genes at all growth phases. The high catalytic efficiency and high transcription levels of L-LDH may provide key explanations for the high optical purity of L-lactic acid produced by *B. coagulans*^12^. There are several *ldhL* genes annotated from the genome sequence in one *B. coagulans* strain while the individual functional role still remains unknown.

In this study, two genes encoding L-LDH (*ldh*L1 and *ldh*L2) were annotated from the whole-genome sequence of *B. coagulans* DSM1. Production of lactic acid and other metabolites was analyzed in both the wild-type and mutant strains. These results provide a first understanding of the roles played by different L-LDHs in thermophilic *B. coagulans* strains and lay the fundament for further engineering this strain to producing other useful bio-chemicals.

## Results

### Characterization of annotated LDH genes in *B. coagulans* DSM1

According to the whole-genome sequence of *B. coagulans* DSM1 (GenBank accession number: CP009709), three possible L-LDH-encoding genes (*ldh*L1, *ldh*L2, and *ldh*L3) and one D-LDH-encoding gene (*ldh*D) were discerned. Using the annotated genome sequence of two related *B. coagulans* strains, 36D1 (GenBank accession number: CP003056) and 2–6 (GenBank accession number: CP002472), as a refs 15, 16, 17, we determined that the enzyme encoded by *ldh*L3 was a leucine dehydrogenase (for strain 36D1) or a glutamate/leucine/phenylalanine/valine dehydrogenase (for strain 2–6). Based on these results, only the first two *ldh*L genes and one *ldh*D gene were chosen for subsequent experiments. After purification of the heterologously expressed His-tagged L-LDHs and D-LDH, the enzyme activities of the purified enzymes were determined. Both L-LDH1 (5.27 ± 0.14 U/mg) and L-LDH2 (4.53 ± 0.26 U/mg) were found to have catalytic activities. Furthermore, the activities of both enzymes were higher than that of D-LDH (1.87 ± 0.08 U/mg), indicating that the activity of L-LDHs in the conversion of pyruvate to L-lactic acid was higher than that of D-LDH in the conversion of pyruvate to D-lactic acid.

**Figure 1:**
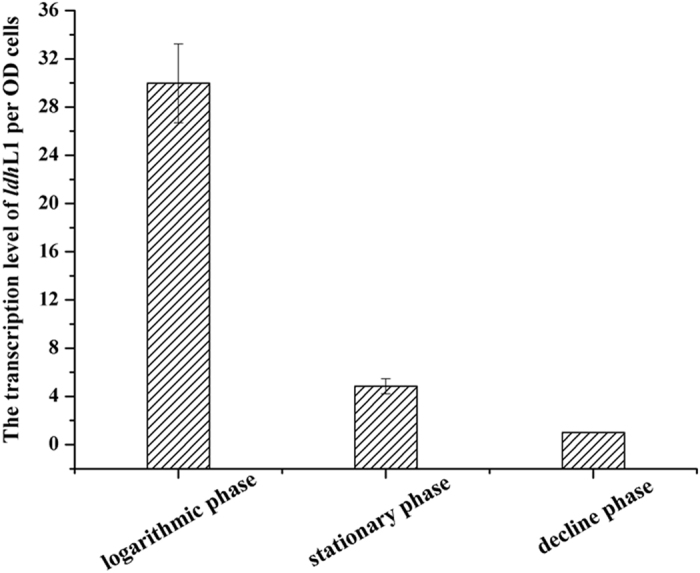
The transcription level of *ldh*L1 per OD cells by RT-PCR analyses. Error bars represent the standard deviations of the means for three independent experiments.

The transcription levels of *ldh*L1, *ldh*L2 and *ldh*D genes were also examined by quantitative real-time (RT)-PCR assay. Although enzymatic activity of L-LDH2 was detected *in vitro*, no transcription level was detected in *ldh*L2 *in vivo*. The transcription ratio of *ldh*L1 to *ldh*D was 80-fold at logarithmic phase, and the transcription levels of *ldh*L1 were higher than those of *ldh*D at different phase (data not shown), which is consistent with the results obtained in *B. coagulans* 2–6^12^. The transcription level of *ldh*L1 per OD cells was also calculated. Results showed that the transcription level of *ldh*L1 was highest at the logarithmic phase and decreased when cell came to stationary phase and decline phase (Fig. 1).

**Figure 2:**
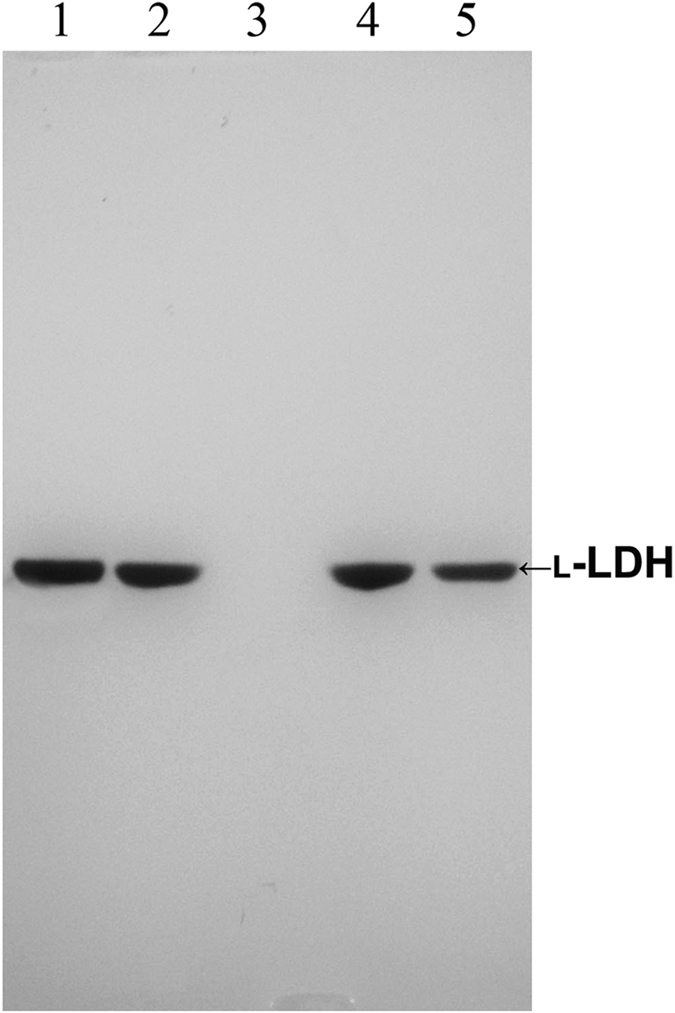
Active staining of *B. coagulans* DSM1 wild-type and mutant strains following native-PAGE. Cell extracts of *B. coagulans* DSM1 (lane 1), DSM1Δ*ldh*L2 (lane 2), DSM1Δ*ldh*L1Δ*ldh*L2 (lane 3), and DSM1Δ*ldh*L1Δ*ldh*L2-*ldh*L1 (lane 4) were used for native-PAGE analysis. DL-Lactate was used as substrates for active staining. Lane 5, the commercial L-LDH enzyme (Sigma-Aldrich) used as a positive control.

Although two L-LDH-encoding genes (*ldh*L1 and *ldh*L2) and one D-LDH-encoding gene (*ldh*D) were annotated in the complete genome of *B. coagulans* DSM1, active staining studies showed that D-LDH activity was not detectable (Fig. 2). Similar results were observed in *B. coagulans* 2–6 of our previous study, in which a D-LDH that catalyzes the production of D-lactic acid has been identified and the activity verified *in vitro*, while contribution of this enzyme to total lactic acid production appears to be minimal^12^. The *in vitro* enzymatic activities and active staining results observed in *B. coagulans* DSM1 were consistent with those of *B. coagulans* 2–6, while the individual role of *ldh*L genes remained unknown in both strains. The genes encoding L-LDH1 (*ldh*L1) was inferred to contribute to L-lactic acid synthesis in *B. coagulans* DSM1. To further determine the roles of two L-LDHs, *ldh*L1and *ldh*L2 were chosen as targets for gene deletion in the subsequent experiments.

### Construction of the *ldh*L2-knockout strain

**Figure 3:**
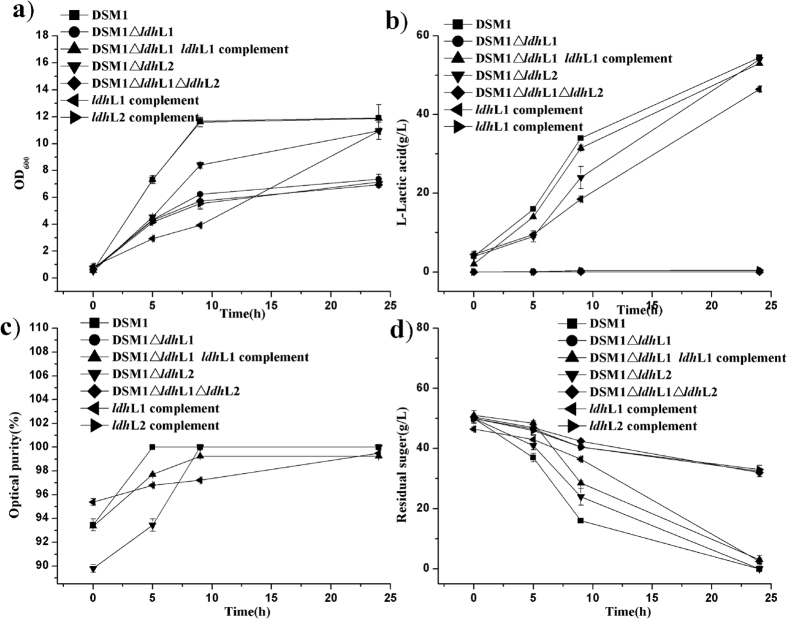
Fermentation profiles of *B. coagulans* DSM1 wild-type and mutant strains. (**a**) A_600_ value, (**b**) L-lactic acid production, (**c**) optical purity of L-lactic acid, and (**d**) glucose consumption. Each data point represents the average of three replicates, with the error bars representing the standard deviation.

As no transcription level of *ldh*L2 was detected *in vivo*, the gene was first knocked out to confirm its role in lactate production. *B. coagulans* DSM1 (wild type) produces L-lactic acid as the primary fermentation product, with an optical purity of 99.8% and a specific productivity of 2.06 g/L/h after subtracting the initial concentration of L-lactate from seed culture (Fig. 3c). Acetic acid, formic acid, and ethanol were not detected in the fermentation broth (Table 1). Although a D-LDH that catalyzes the production of D-lactic acid has been identified and expressed in *E. coli*, the contribution of this enzyme to the total lactic acid produced appeared to be minimal in *B. coagulans* DSM1, since only trace amount (<0.1 g/L) of D-lactate was detected in the broth. Fermentation profile of DSM1Δ*ldh*L2 was the same as that observed for the wild-type strain, except that the growth rate of DSM1Δ*ldh*L2 was slightly slower than the parent strain (Fig. 3a), and trace amounts of acetic acid (0.39 g/L) were detected (Table 1). The same L-lactic acid optical purity (99.8%), with a consistent specific productivity of 2.04 g/L/h, was obtained in the fermentation broth of DSM1Δ*ldh*L2, indicating that *ldh*L2 plays a minimal role in L-lactic acid produced by *B. coagulans* DSM1.

### Construction of the *ldh*L1-knockout and *ldh*L1 *ldh*L2 double deletion strains

Deletion of *ldh*L1 resulted in significantly reduced cell-growth rates to value ~50% of those observed in the wild-type strain (Fig. 3a), and only 34% glucose was consumed by the DSM1Δ*ldh*L1 strain (Fig. 3d). Small amounts of L-lactic acid (0.17 g/L), with a productivity of 0.01 g/L/h, were detected. Compared with the wild type strain, ethanol (1.40 g/L), formic acid (1.22 g/L) and acetic acid (1.13 g/L) were produced by DSM1Δ*ldh*L1 (Table 1). Double deletion of gene *ldh*L1*ldh*L2 (DSM1Δ*ldh*L1*ldh*L2) significantly reduced cell-growth rates and glucose consumption, which was the same situation with strain DSM1Δ*ldh*L1 (Fig. 3a and d). No L-lactic acid was detected in the fermentation broth, while acetic acid (2.54 g/L) become the major product, with only a little amount of D-lactic acid (0.26 g/L) present. The main product shifted from lactic acid to acetic acid, formic acid, and ethanol, as well as increased concentration of pyruvate as compared with DSM1 and DSM1Δ*ldh*L2 results (Table 1). These results indicatied that *ldh*L1 plays a central role in producing optically pure L-lactic acid in *B. coagulans* DSM1.

### *Ldh*L1 is essential for optically pure L-lactic acid synthesis in strain DSM1

To determine whether *ldh*L1 is the key factor affecting L-lactic acid production in *B. coagulans* DSM1, *ldh*L-complemented strains (DSM1Δ*ldh*L1-*ldh*L1, DSM1Δ*ldh*L1Δ*ldh*L2-*ldh*L1 and DSM1Δ*ldh*L1Δ*ldh*L2-*ldh*L2) were constructed. Complementation of gene *ldh*L1 (DSM1Δ*ldh*L1-*ldh*L1 and DSM1Δ*ldh*L1Δ*ldh*L2-*ldh*L1) with its native promoter expressed in plasmid pNW33n restored cell growth and L-lactic acid production profiles to wild-type level (Fig. 3). The main products of two *ldh*L1 complemented strains were shifted back to L-lactic acid with an optical purity of 99.4% and specific productivities of 2.01 g/L/h and 2.10 g/L/h, which is consistent with that of the wild-type. Although trace amounts of acetic acid and ethanol were detected by HPLC, the amounts of formic acid were reduced to values below the detection limit. Notably, the concentration of D-lactic acid also decreased to the consistent level with the wild-type (Table 1). Meanwhile, *ldh*L2 complementation resulted in no significant change in glucose consumption and cell growth compared with double knockout strain (DSM1Δ*ldh*L1Δ*ldh*L2), except that small amounts of L-lactic acid (0.49 g/L) was produced. Complementing the double mutant with *ldh*L2 did not lead to high L-lactic acid production as the wild type strain.

Enzyme expression in cell extracts were also confirmed by active staining of LDHs after native-PAGE. As expected, L-LDH activity was detected in *B. coagulans* DSM1 and native-PAGE showed that L-LDH activity was still detected in *B. coagulans* DSM1Δ*ldh*L2 (Fig. 2). Further gene deletion of Δ*ldh*L1 resulted in no detectable L-LDH activity according to native-PAGE results, while L-LDH activity was detected in the *ldh*L1-complementary strain (DSM1Δ*ldh*L1Δ*ldh*L2-*ldh*L1). These results showed that although two L-LDH-encoding genes were annotated in the *B. coagulans* DSM1 genome, the catalytic efficiency of the enzyme encoded by *ldh*L1 involving L-lactic acid production was much higher than that observed in the enzyme encoded by *ldh*L2. For all strains, only one fragment corresponding to L-LDH activity was observed when gels were soaked with DL-lactate, indicating that only L-LDH activities were detected in the *B. coagulans* DSM1 strains, and that D-LDH activity might be too low to be detected in all the strains^12^.

## Discussion

*B. coagulans* DSM1 is a homofermentative L-lactic acid producer, with a high optical purity of 99.8%. L-nLDH is responsible for the synthesis of L-lactic acid. However, two L-LDH-encoding genes (*ldh*L1 and *ldh*L2) and one D-LDH-encoding gene (*ldh*D) were annotated in the complete genome of *B. coagulans* DSM1. Similar results were observed in other *B. coagulans* strain^12^. The individual role of these genes in L-lactic acid production remained unknown. Thus, the genes responsible for L-lactic acid production and their mechanisms were the focus of this study.

Construction of a targeted gene deletion system is one of the most effective approaches for analyzing gene function *in vivo*. As the genetic manipulation of parent strain, such as *B. coagulans* 2–6, is currently not available, its phylogenetically close type strain *B. coagulans* DSM1 was used in this study since the genome sequence of *B. coagulans* 2–6 shares a high similarity (>99%) with *B. coagulans* DSM1^15^,^16^. Deletion of *ldh*L1 led to alteration of the main product of *B. coagulans* DSM1 from L-lactic acid to acetic acid and other byproducts. Compared with *ldh*L2, *ldh*L1 plays a central role in producing optically pure L-lactic acid in *B. coagulans* DSM1.

LDHs play a complex role in *B. coagulans* by not only catalyzing pyruvate transformation to lactic acid, but also by catalyzing NADH oxidation. As a predominant redox product of catabolism, NADH plays an important role in over 700 biochemical reactions, a number of which constitute synthetically practical enzymatic reactions^18^. Under micro-aerobic conditions, NADH generated from glycolysis is mainly consumed by two metabolic pathways: the lactate-production pathway and the alcohol-production pathway^19^. The primary product of glucose fermentation in *B. coagulans* DSM1 is L-lactic acid (~98.5% of the fermentation products), indicating that the lactic acid-production pathway constitutes the main NADH-metabolism pathway in *B. coagulans* DSM1. The physiological role of LDHs in bacteria is to balance regeneration of NAD^+^ during fermentation, which is an important step in the metabolism and energy conversion of living cells, since it allows re-oxidation of NADH, which is necessary for glycolysis. In this study, *ldh*L1 knockout resulted in the complete loss of LDH function affecting intracellular NADH distribution and eliminating the primary route for NADH oxidation. The redox imbalance that resulted from the elimination of the main NAD^+^-regeneration pathway affected cellular metabolism and thus impaired growth rates^20^,^21^.

To maintain cellular redox balance, pyruvate produced from the glycolytic breakdown of carbohydrates would be metabolized by several alternative pathways in the absence of active LDH^22^. The common NADH-oxidation pathway in *Gluconobacter oxydans* involves pyruvate oxidation to acetyl-coenzyme A (CoA), followed by: 1) CoA entry into the TCA cycle, which provides carbon and energy resources for bacterial growth; or 2) accumulated pyruvate entry into a non-energy-generating pathway, with acetate as the final product^23^. It was also suggested that accumulation of intracellular NADH might enhance hydrogen production by a putative membrane-bound hydrogenase in *Enterobacter aerogenes*^19^. In this study, *ldh*L mutants exhibited increased yields of acetic acid as compared to the wild-type strain, which showed no acetic acid production (Table 1). The acetic acid yield of the DSM1Δ*ldh*L1Δ*ldh*L2 strain (2.54 g/L) was significantly higher than that of the DSM1Δ*ldh*L2 strain (0.39 g/L). Compared with *G. oxydans, B. coagulans* DSM1 preferred to use an energy-generating pathway, with acetic acid production, to metabolize the accumulated NADH following loss of LDH function, because acetate kinase encoding gene exists in *B. coagulans* DSM1 genome. Formic acid and ethanol were the other products accumulated in the DSM1Δ*ldh*L1Δ*ldh*L2. Formic acid is produced by pyruvate formate lyase (PFL). In order to maintain redox balance, the PFL-produced acetyl-CoA needs to be converted to acetate and ethanol, which could well explain the product alteration in the LDH null strain (Figure S1 in Supporting Information)^24^. Furthermore, some glucose was consumed for CoA production, which entry into the TCA cycle for bacterial growth. The DSM1Δ*ldh*L1Δ*ldh*L2 strain also produced increased amount of D-lactic acid (0.26 g/L) that were twice higher than the values in wild-type strain or DSM1Δ*ldh*L2. One possible explanation is that the elimination of the main NAD^+^-regeneration pathway and the apparent lack of an alternative pathway in the DSM1Δ*ldh*L1Δ*ldh*L2 strain provoked the increased expression of other NADH consumption enzymes, such as D-LDH^25^.

In conclusion, two L-LDH-encoding genes (*ldh*L1 and *ldh*L2) were identified in the *B. coagulans* DSM1 genome. Although the functional role of *ldh*L2 needs to be further investigated, the results obtained in this study confirmed that only *ldh*L1 is the key encoded gene enabling L-LDH activity and L-lactic acid production in *B. coagulans* DSM1. This study provides useful information for the further engineering of *B. coagulans* DSM1 into a platform for production of other value-added chemicals.

## Methods

### Bacterial strains, plasmids, and culture conditions

The bacterial strains and plasmids used in this study are listed in Table S1 in Supporting Information. As the genetic manipulation of strain *B. coagulans* 2–6 is currently not available, its phylogenetically close type strain *B. coagulans* DSM1 was used in this study^15^,^16^. *B. coagulans* DSM1 is a homofermentative producer of L-lactic acid to an optical purity of 99.8%, which was purchased from Deutsche Sammlung von Mikroorganismen und Zellkulturen GmbH (DSMZ, Braunschweig, Germany). For genetic engineering, *B. coagulans* DSM1 was grown in BC medium at 45 °C, 120 rpm^26^. For lactic acid fermentation, *B. coagulans* DSM1 was grown in glucose yeast-extract carbonate (GYC) medium (50 g glucose, 10 g yeast extract, and 30 g CaCO_3_ per liter) at 50 °C, 120 rpm^9^. The pH value was maintained at 6.2~6.5 with the addition of CaCO_3_ and the inoculum volume was 10% (v/v). *Escherichia coli* was grown aerobically in Luria-Bertani (LB) medium at 37 °C, 200 rpm. Kanamycin (Kan) and chloramphenicol (Cm) were added to the medium when required at concentrations of 40 μg/mL Kan for *E. coli*, and 7 μg/mL and 25 μg/mL Cm for *B. coagulans* DSM1 and *E. coli*, respectively.

### Cloning, expression, and assay of enzymes responsible for lactic acid production

Homologous genes encoding both L-LDH (*ldh*L1 and *ldh*L2) and D-LDH (*ldh*D) were amplified by polymerase chain reaction (PCR) from *B. coagulans* DSM1 genomic DNA using *Pfu* DNA polymerase (Takara Bio Co., Dalian, China). The primers are listed in Table S2 in Supporting Information. Three DNA fragments were cloned into the pET-28a expression vector, and the recombinant plasmids were transformed into competent *E. coli* BL21 (DE3) cells, respectively. For protein expression, the cells were grown to an optical density of A_600_ = 0.6–0.8 at 37 °C, then induced with 0.5 mM isopropyl-*β*-d-1- thiogalactopyranoside and grown for an additional 12 h at 18 °C.

The purification procedures and activity assays of the enzymes were employed as previously described^12^. Enzymatic assays were performed in transparent 96-well plates, and the reaction mixture containing 100 mM sodium phosphate (pH 6.5), 200 μM NADH, and 0.1 mg/ml enzyme. The mixture was pre-incubated at 50 °C for 10 min. To start the reaction, sodium pyruvate was added to a final concentration of 20 mM, and NADH oxidation was monitored at 340 nm in a 96-well plate reader. One unit (1 U) of LDH activity was defined as the amount of enzyme required to reduce 1 μmol nicotinamide adenine dinucleotide (NAD) per minute.

### Transcriptional analysis of lactate dehydrogenase gene expression

Determination of the transcription levels of *ldh*L1, *ldh*L2 and *ldh*D genes was analyzed by quantitative RT-PCR according to the literature^27^. Initially, *B. coagulans* DSM1 were inoculated in GYC broth at 50 °C to measure the growth curve and time courses of optical purity during fermentation for the determination of sample points. Cells of the three representative strains were harvested by centrifugation (5,000 × *g* for 10 min, 4 °C) for RNA isolation by using an E.Z.N.A.^TM^ Bacterial RNA Kit (Omega). Total RNA concentration was determined from the absorbance at 260 nm (NanoVue, GE). cDNA copies were synthesized with a FastQuant RT Kit (with gDNase) (Tiangen, China) and amplified with SYBR Premix Ex Taq (TaKaRa, China). The threshold cycle for each PCR with different concentrations of cDNA was determined and compared against a standard DNA (16 S rRNA gene) that was also analyzed at the same time. The 2^−△△Ct^ relative quantification method was used to determine the mRNA levels.

### Construction of *B. coagulans* DSM1 *ldh*L gene knockout mutants

The *ldh*L gene knockouts were performed by using plasmid pMH77 with the thermosensitive lactococcal pSH71/pWV01 replicon and the procedures were conducted according to the reference 26. The primers used for gene deletion are listed in Table S2 in Supporting Information. For knocking out *ldh*L2 gene from DSM1, the flanking regions of the *ldh*L2 gene were first PCR amplified from *B. coagulans* DSM1 genomic DNA using the primers L2 up-For and L2 up-Rev (upstream, 1000 bp) and L2 down-For and L2 down-Rev (downstream, 1000 bp), respectively. After gel purification, overlap extension PCR was performed, wherein the upstream and downstream regions were fused using the primers L2 up-For and L2 down-Rev. The resulting PCR product was gel purified, digested with EcoRI and XhoI, and cloned into pMH77, resulting in plasmid pMH77-Δ*ldh*L2. Plasmid pMH77-Δ*ldh*L2 was first transformed into *Lactococcus lactis* MG1363. And then the extracted plasmid pMH77-Δ*ldh*L2 was transformed into *B. coagulans* DSM1 by electroporation.

For integration, a colony harboring the integration plasmid was cultured overnight at 45 °C, followed by a temperature shift to 60 °C and further incubation for 12 h. A dilution series was plated on BC plates containing Cm and incubated at 60 °C until colonies appeared. Colonies were picked and tested for first single crossover recombination by PCR analysis. Then the right colonies were incubated in BC liquid broth without Cm overnight at 45 °C, 120 rpm, and then a dilution series was streaked onto BC plates without Cm and incubated overnight at 45 °C. Colonies were sequentially streaked onto BC plates with and without Cm and incubated overnight at 45 °C. The colonies that grew on BC plates without Cm, but did not grow on those with Cm, were picked out for PCR analysis using the primers L2 For and L2 Rev.

For knocking out *ldh*L1 gene, the involved steps are similar to those described for DSM1Δ*ldh*L2. The *ldh*L1-flanking regions were first PCR amplified from *B. coagulans* DSM1 genomic DNA using the primers L1 up-For and L1 up-Rev (upstream, 1000 bp) and L1 down-For and L1 down-Rev (downstream, 1000 bp), respectively. After gel purification, overlap extension PCR was performed, wherein the upstream and downstream regions were fused using the primers L1 up-For and L1 down-Rev. Then, the *ldh*L1-flanking regions were cloned into the integration vector to construct the plasmid of pMH77-Δ*ldh*L1, and introduced into *B. coagulans* DSM1 by electroporation to construct the double gene-deletion strain (DSM1Δ*ldh*L1). The gene knockout mutant was confirmed by PCR using the primer pairs L1 For/L1 Rev.

The involved steps of *ldh*L1*ldh*L2 gene double deletion are similar to those described for DSM1Δ*ldh*L1. Plasmid pMH77-Δ*ldh*L1 was introduced into *B. coagulans* DSM1Δ*ldh*L2 by electroporation to construct the double gene-deletion strain (DSM1Δ*ldh*L1Δ*ldh*L2). The gene knockout mutant was confirmed by PCR using the primer pairs L1 For/L1 Rev.

### Construction of *ldh*L complementation strain

For construction of the *ldh*L1 complementation plasmid pNW33n-*ldh*L1, the *ldh*L1 gene with its native promoter (1182-bp upstream of the gene) was amplified from *B. coagulans* DSM1 genomic DNA using the primers *ldh*L1-For and *ldh*L1-Rev (Table S2 in Supporting Information). The fragment was cloned into pNW33n by using the restriction sites of HindIII and SacI. The resulting plasmid (pNW33n-*ldh*L1) was introduced into *B. coagulans* DSM1Δ*ldh*L1 and *B. coagulans* DSM1Δ*ldh*L1Δ*ldh*L2 by electroporation to construct the *ldh*L1-complemented strains.

For construction of the *ldh*L2 complementation plasmid pNW33n-*ldh*L2, the *ldh*L2 gene with its native promoter sequence was amplified from *B. coagulans* DSM1 genomic DNA using the primers *ldh*L2-For and *ldh*L2-Rev (Table S2 in Supporting Information). The fragment was cloned into pNW33n, and the resulted plasmid (pNW33n-*ldh*L2) was introduced into *B. coagulans* DSM1Δ*ldh*L1Δ*ldh*L2 by electroporation to construct the *ldh*L2-complemented strain.

### Active staining of L-LDHs

*B. coagulans* DSM1 wild-type, DSM1Δ*ldh*L2, DSM1Δ*ldh*L1Δ*ldh*L2, and DSM1 *ldh*L1-complementation strains were grown in 50 mL GYC medium to logarithmic phase at 50 °C, 120 rpm. Exponentially growing cells were harvested by centrifugation (10,540 *g*, 10 min, 4 °C) and washed with 0.85% (w/v) physiological saline. Cell pellets were subsequently suspended in 100 mM potassium phosphate buffer (pH 7.0) and disrupted by sonication in an ice bath. After centrifugation at 12,000 *g* for 10 min, the supernatants were used as the crude enzymes.

Active staining of L-LDHs was performed according to a previous report with some modifications^12^. Briefly, crude enzymes of the four representative strains were concentrated by ultrafiltration. and separated by native-polyacrylamide gel electrophoresis (PAGE) on gradient 4–20% native polyacrylamide gels. Protein concentrations were determined with a Bradford protein assay kit (Bio-Rad). Loading quantity of each sample was 5 μg. After electrophoresis, gels were soaked with 100 mM Tris-HCl buffer (pH 8.0) containing 0.1 mM phenazinemethosulfate, 0.1 mM nitrotetrazolium blue chloride, 2 mM NAD, and 100 mM DL-lactate.

### Analytical methods

The growth curves were measured at A_600_ by a 7200 Visible Spectrophotometer (UNICO, Shanghai, China). L-Lactic acid concentrations were measured using a SBA-40C biosensor analyzer (Institute of Biology, Shandong Academy of Sciences, China). The optical purity of L-lactic acid was analyzed using high-performance liquid chromatography (HPLC, Agilent 1260 Series, Hewlett-Packard, Palo Alto, CA, USA) equipped with a chiral column (MCI GEL CRS10W, Tokyo, Japan)^28^. The mobile phase consisted of 2 mM CuSO_4_ at a flow rate of 0.5 mL/min (25 °C), with UV detection at 254 nm. The optical purity of L-lactic acid was defined as [L-lactic acid/(L-lactic acid + D-lactic acid) × 100%]. Glucose, ethanol and organic acids (oxaloacetic acid, formic acid, acetic acid, pyruvic acid, maletate acid and lactic acid) were measured at 210 nm by using an HPLC system equipped with an organic-acid column (MCI GEL CRS10W) with a UV and differential detector. The mobile phase consisted of 6 mM H_2_SO_4_ at a flow rate of 0.5 mL/min (55 °C).

## Additional Information

**How to cite this article**: Sun, L. *et al*. Contributory roles of two L-lactate dehydrogenases for L-lactic acid production in thermotolerant *Bacillus coagulans. Sci. Rep.* **6**, 37916; doi: 10.1038/srep37916 (2016).

**Publisher’s note:** Springer Nature remains neutral with regard to jurisdictional claims in published maps and institutional affiliations.

## Supplementary Material

Supporting Information

## Figures and Tables

**Table 1:** Metabolite characteristics of *B. coagulans* DSM1wild-type and mutant strains.

Products	DSM1	DSM1△*ldh*L1	DSM1△*ldh*L1 -*ldh*L1	DSM1△*ldh*L2	DSM1△*ldh*L1△*ldh*L2	DSM1△*ldh*L1△*ldh*L2-*ldh*L1	DSM1△*ldh*L1△*ldh*L2-*ldh*L2
L-Lactic acid (g/L)	49.40 ± 0.56	0.17 ± 0.01	50.39 ± 0.37	48.9 ± 0.38	ND	48.22 ± 0.32	0.49 ± 0.01
Productivity of L-lactic acid (g/L/h)	2.06 ± 0.02	0.01 ± 0	2.10 ± 0.01	2.04 ± 0.02	ND	2.01 ± 0.01	0.02 ± 0
D-Lactic acid (g/L)	0.10 ± 0	0.32 ± 0.01	0.39 ± 0.01	0.10 ± 0	0.26 ± 0.01	0.17 ± 0	0.65 ± 0.03
Ethanol (g/L)	ND^a^	1.40 ± 0.03	0.80 ± 0.04	ND	0.19 ± 0.01	ND	0.99 ± 0.05
Oxaloacetic acid (g/L)	ND	ND	ND	ND	0.02 ± 0	ND	ND
Formic acid (g/L)	ND	1.22 ± 0.09	ND	ND	1.14±0.02	ND	1.26 ± 0.08
Acetic acid (g/L)	ND	1.13 ± 0.12	0.90 ± 0.06	0.39 ± 0.03	2.54 ± 0.23	1.89 ± 0.09	1.08 ± 0.02
Pyruvic acid (g/L)	1.25 ± 0.12	1.01 ± 0.04	0.22 ± 0.03	ND	1.98 ± 0.11	0.21 ± 0.03	1.23 ± 0.03

^a^ND, not detected.
