# Supplementary material for: Contributory roles of two l-lactate dehydrogenases for l-lactic acid production in thermotolerant Bacillus coagulans
Source: Sci Rep. 2016 Nov 25;6:37916. doi: 10.1038/srep37916 (PMC5122838; doi:10.1038/srep37916)
Supplement: Supporting Information [file srep37916-s1.doc]

**Supporting information for**

**Contributory roles of two l-lactate dehydrogenases for l-lactic acid production in thermotolerant *Bacillus coagulans***

Lifan Sun^a,b†^, Caili Zhang^a†^, Pengcheng Lv^c^, Yanping Wang^b^, Limin Wang^a*^ , Bo Yu^a,*^

^a^ *CAS Key Laboratory of Microbial Physiological and Metabolic Engineering, Institute of Microbiology, Chinese Academy of Sciences, Beijing 100101, China*

^b^ *School of Food Engineering and Biological Technology, Tianjin University of Science & Technology, Tianjin 300457, China*

^c^ *College of Life Science and Bioengineering, Beijing University of Technology, Beijing 100124, China*

^†^ L.S. and C.Z. contributed equally to this study.

* Corresponding author.

Correspondence and requests for materials should be addressed to L. W. ([wanglimin@im.ac.cn](mailto:wanglimin@im.ac.cn)) or B. Y. ([yub@im.ac.cn](mailto:yub@im.ac.cn)) .

Phone/Fax: +86-10-6480 6132

Lifan Sun, *E-mail*: [1102108321@qq.com](mailto:1102108321@qq.com)

Caili Zhang, *E-mail*: [cailizhang2012@163.com](mailto:cailizhang2012@163.com)

Yanping Wang, *E-mail*: [ypwang40@163.com](mailto:ypwang40@163.com)

Pengcheng Lv, , *E-mail*: [lpc1995320@163.com](mailto:lpc1995320@163.com)

**Table S1** Strains and plasmids used in this study.

| Strain or plasmid | Relevant features ^a^ | Reference or source |
| --- | --- | --- |
| *Strains* |  |  |
| *B. coagulans* strains  DSM1  DSM1Δ*ldh*L1  DSM1Δ*ldh*L1- *ldh*L1  DSM1Δ*ldh*L2  DSM1Δ*ldh*L1Δ*ldh*L2  DSM1Δ*ldh*L1Δ*ldh*L2- *ldh*L1  DSM1Δ*ldh*L1Δ*ldh*L2- *ldh*L2  *E. coli* strains  BL21(DE3)  DH5α | Wild type |  |
|  |  | DSMZ, Germany |
|  | *ldh*L1 null mutant  Complementation of DSM1Δ*ldh*L1 with *ldh*L1  *ldh*L2 null mutant  *ldh*L1and *ldh*L2 null mutant | This study  This study  This study |
|  |  | This study |
|  | Complementation of DSM1Δ*ldh*L1Δ*ldh*L2 with *ldh*L1  Complementation of DSM1Δ*ldh*L1Δ*ldh*L2 with *ldh*L2 | This study |
|  |  | This study |
|  | Host for protein expression  Host for gene cloning |  |
|  |  | Tiangen Co., China  Tiangen Co., China |
| *Plasmids*  pET-28a  pET-28a-*ldh*L1  pET-28a-*ldh*L2  pET-28a-*ldh*D  pUC19 | Protein expression vector, Kan^r^ |  |
|  |  | Merck Co., Germany |
|  | N-terminal His-tagged *ldh*L1 in pET28a, Kan^r^  N-terminal His-tagged *ldh*L2 in pET28a, Kan^r^ | This study |
|  |  | This study |
|  | N-terminal His-tagged *ldh*D in pET28a, Kan^r^  Cloning vector, Ap^r^ | This study |
|  |  | Tiangen Co., China |
| pMH77  pNW33n  pNW33n-*ldh*L1  pNW33n-*ldh*L2  pMH77-Δ*ldh*L1  pMH77-Δ*ldh*L2 | pSH71 replication containing temperature sensitive vector, Cm^r^ | 25 |
|  |  |  |
|  | *E. coli*-*Bacillus* shuttle vector, cloning vector, Cm^r^  *ldh*L1-restoration vector, pNW33n harboring *ldh*L1gene with its native promoter, Cm^r^  *ldh*L2-restoration vector, pNW33n harboring *ldh*L2 gene with its native promoter, Cm^r^  *ldh*L1 gene deletion vector, Cm^r^ | BGSC, USA |
|  |  | This study  This study  This study |
|  | *ldh*L2 gene deletion vector, Cm^r^ | This study |

^a^ *Cm^r^* chloramphenicol resistant, *Kan^r^* kanamycin resistant, *Ap^r^* ampicillin resistant.

**Table S2** Primers used in this study.

| Primer | Sequence 5′→ 3′^a^ |
| --- | --- |
| *Primers used for gene expression in pET-28a* | |
| DSM1*ldh*L1F | CCGGAATTCATGAAAAAAGTCAATCGTATTGC |
| DSM1*ldh*L1R | CCGCTCGAGTTACAATATCGGTGCCATTGTTTC |
| DSM1*ldh*L2F | TTCCATATGATGAGAAAGACGAAATTGGTGGTTG |
| DSM1*ldh*L2R | CCGCTCGAGAGCCCGAATATACGATTTTCCGG |
| DSM1*ldh*DF | CGCGGATCCATGAGAAAAGTTGTTGCC |
| DSM1*ldh*DR | CCGCTCGAGTACTTTTATCTCCCACCTG |
|  |  |
| *Primers used for gene deletion* | |
| L1 up-For | CCGGAATTCGCTCCTTTCATTTGGTCAGAAAAATG |
| L1 up-Rev | AGCCCGGCCGGCACAAATGCATATAATCTTCCTCCCCATC |
| L1 down-For | GATGGGGAGGAAGATTATATGCATTTGTGCCGGCCGGGC |
| L1 down-rev | CCGCTCGAGGATCAACCGGGTCAGTGCAGTC |
| L1 For  L1 Rev | GGGGGGCTTTCTTTTCATCAATTTG  TGATTGAAACGATTTTAAACGCGG |
| L2 up-For | CCGGAATTCTGAAGGAGGGATACATATTTG |
| L2 up-Rev | GCCCTTTTACACCTTTCAGGTGTTGTCTCCCCTCCTTGTTTTC |
| L2 down-For | AACAAGGAGGGGAGACAACACCTGAAAGGTGTAAAAGGGC |
| L2 down-Rev | CCGCTCGAGCGGAGCACTGACATCGCAATACGAT |
| L2 For  L2 Rev | AAACCGTCAACCGGGTTGTATTCCAGG  GATGATGATCATCAAAATAAACATCAAACCGGC |
|  |  |
| *Primers used for gene complementation* | |
| *ldh*L1-For | CCCAAGCTTAGCCTCATCGCCGGTTTCCCTCGC |
| *ldh*L1-Rev  *ldh*L2-For  *ldh*L2-Rev | CGCGAGCTCTTACAATATCGGTGCCATTGTTTCT  CCCAAGCTTTGGGCGATGCCAATCTGGCTTTATG  CGAGCTCTCAAGCCCGAATATACGATTTTCCG |

^a^ Restriction sites in the primer sequences are underlined.

**
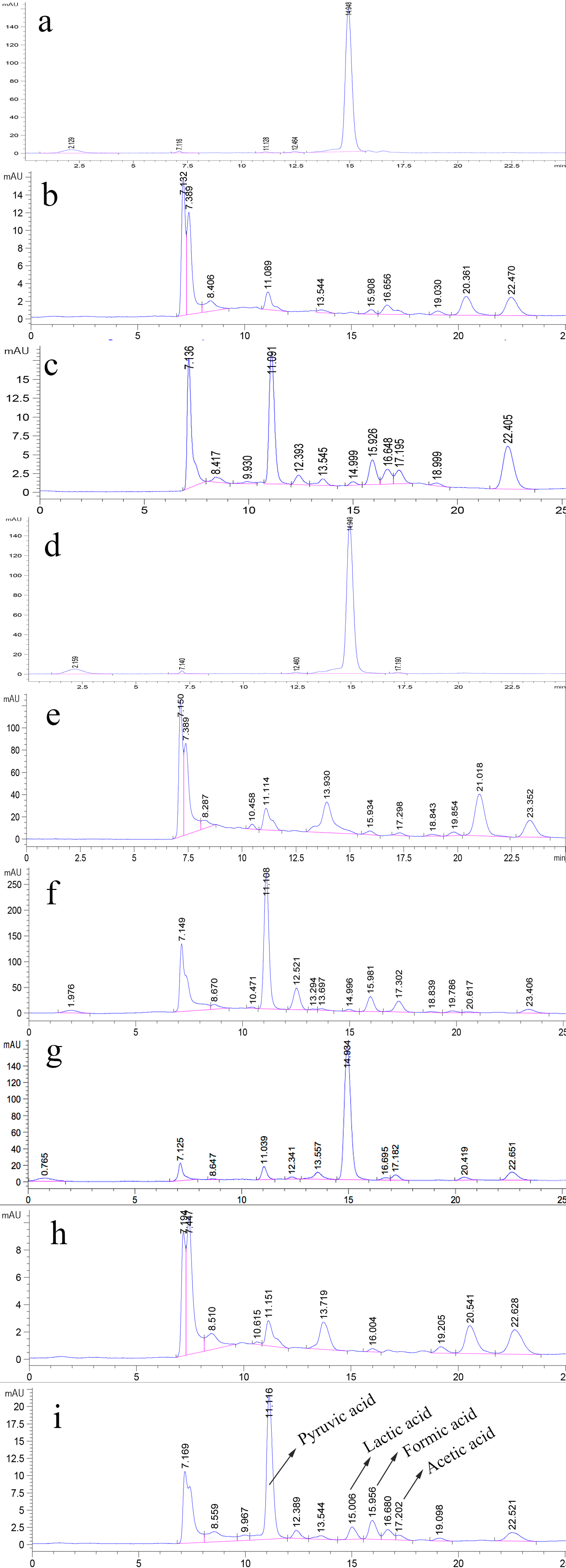
**

**Figure** **S1**. **HPLC analysis of fermentation products in *Bacillus coagulans* DSM1 wild-type strain and mutants.**

DSM1 at 24-h (a), DSM1Δ*ldh*L1 at 0-h (b), DSM1Δ*ldh*L1 at 24-h (c), DSM1Δ*ldh*L2 at 24-h (d), DSM1△*ldh*L1△*ldh*L2 at 0 h (e), DSM1△*ldh*L1△*ldh*L2 at 24-h (f), DSM1△*ldh*L1△*ldh*L2-*ldh*L1 complementation at 24-h (g), DSM1△*ldh*L1△*ldh*L2-*ldh*L2 complementation at 0-h (h), DSM1△*ldh*L1△*ldh*L2-*ldh*L2 complementation at 24-h (i).
